# Balanced Crystalloids Versus Normal Saline for Fluid Resuscitation in Patients With Diabetic Ketoacidosis: A Systematic Review

**DOI:** 10.7759/cureus.113199

**Published:** 2026-07-22

**Authors:** Mohamed Ahmed Hamed, Talal Abdullah Ahmed Alnajjar, Ziyad Daifallah Alsubhi

**Affiliations:** 1 Emergency Medicine, Alhada Armed Forces Hospital, Taif, SAU

**Keywords:** 0.9% saline, balanced crystalloids, diabetic ketoacidosis, dka, lactated ringer's, normal saline, plasma-lyte, ringer's lactate, sterofundin

## Abstract

Diabetic ketoacidosis (DKA) is a life-threatening metabolic complication of diabetes mellitus requiring aggressive intravenous fluid resuscitation. Although the recommended fluid has always been 0.9% normal saline (NS), its supraphysiological chloride load may induce hyperchloremic metabolic acidosis, potentially complicating DKA management. Balanced crystalloids (BCs), with electrolyte compositions more closely approximating human plasma, have emerged as potentially advantageous alternatives. This systematic review compares the clinical outcomes of NS with BCs for fluid resuscitation in patients with DKA.

A comprehensive literature search was conducted in PubMed/MEDLINE, Scopus, Embase, and CENTRAL (2021-2026) following PRISMA guidelines. Studies that directly compared 0.9% NS with BCs (such as lactated Ringer's (LR) solution, Plasma-Lyte 148, Isolyte, Sterofundin, or other balanced electrolyte solutions (BES)) for DKA resuscitation were included. The outcomes of interest included time to DKA resolution, electrolyte profiles, insulin infusion duration, length of stay (LOS), acute kidney injury (AKI), and mortality. The risk of bias (ROB) was assessed using the Risk of Bias in Non-randomized Studies of Interventions (ROBINS-I) (for non-randomized studies) and RoB 2 (for randomized controlled trials (RCTs)).

Nine studies, four RCTs and five non-randomized studies, comprising 1416 patients, met the inclusion criteria. BCs consistently reduced hyperchloremia and improved bicarbonate profiles compared with NS. Insulin infusion duration was significantly shorter with BCs in two studies. Length-of-stay outcomes were mixed, with some studies showing shorter hospital or ICU stays with BCs. No significant differences in AKI incidence or mortality were observed between groups. The ROB ranged from moderate to substantial, although the RCTs demonstrated a lower ROB than the retrospective cohort studies. BCs demonstrated superior electrolyte profiles and comparable safety compared with NS in DKA. However, their effect on clinically meaningful outcomes such as time to DKA resolution remains uncertain. While the biochemical advantages support consideration of BC, definitive practice recommendations await higher-certainty evidence.

## Introduction and background

Diabetic ketoacidosis (DKA) is one of the most serious acute metabolic complications of diabetes mellitus, characterized by hyperglycemia, metabolic acidosis, and ketonemia. The global burden of DKA is substantial, with an estimated annual incidence of 4% to 8% among adults with type 1 diabetes mellitus. Furthermore, up to 80% of children and adolescents under 15 years of age present with DKA at the time of their initial type 1 diabetes diagnosis [1]. In industrialized nations, mortality rates range from 2% to 5%, whereas in developing countries, mortality can reach 6% to 24%, highlighting persistent gaps in acute diabetes care worldwide [2]. The cornerstone of DKA management encompasses three interdependent components: (1) aggressive intravenous fluid resuscitation to restore intravascular volume; (2) continuous intravenous insulin infusion to suppress ketogenesis and normalize blood glucose; and (3) meticulous electrolyte monitoring with appropriate replacement. Among these, fluid resuscitation is arguably the most critical initial intervention; however, the optimal choice of crystalloid solution for this purpose remains a subject of considerable clinical debate [2].

Historically, 0.9% normal saline (NS) has been the preferred fluid for DKA resuscitation, being a hypertonic solution with sodium and chloride concentrations of 154 mmol/L. This recommendation has been perpetuated by major clinical practice guidelines for decades [3]. However, the administration of large volumes of NS (often 4 to 8 liters over the first 24 hours of DKA treatment) carries the well-recognized iatrogenic risk of inducing hyperchloremic metabolic acidosis [1]. This occurs because the supraphysiological chloride load provided by NS exceeds the kidneys' capacity for chloride excretion, resulting in a non-anion gap metabolic acidosis that can superimpose upon the pre-existing elevated anion gap acidosis of DKA. Such hyperchloremia may obscure the true resolution of DKA, as clinicians may continue insulin infusion based on a persistently low bicarbonate level that is actually driven by chloride rather than ongoing ketogenesis. Furthermore, hyperchloremia has been associated with renal vasoconstriction, reduced glomerular filtration rate, and a higher risk of acute kidney injury (AKI) in critically ill patients, raising legitimate concerns about saline's safety as the standard resuscitation fluid in DKA [4].

Balanced crystalloid (BCs) solutions, such as Ringer's lactate (RL), Plasma-Lyte 148, Isolene, and Sterofundin, have surfaced as potentially beneficial substitutes to deal with these problems. BCs are designed with electrolyte compositions more closely approximating that of human plasma, typically containing chloride concentrations between 98 and 113 mmol/L, which is substantially lower than that of NS. The theoretical advantages of BCs in DKA are compelling: by avoiding an excessive chloride load, they may prevent or attenuate the development of iatrogenic hyperchloremic acidosis, thereby allowing the anion gap to serve as a more reliable marker of true metabolic resolution. Additionally, BCs may limit the risk of AKI, shorten the duration of insulin infusion, reduce the amount of fluid needed for resuscitation, and shorten the length of stay (LOS) in the intensive care unit [5]. However, lingering concerns regarding the lactate content of some balanced solutions (typically 28 mmol/L in lactated Ringer's (LR)) and the theoretical risk of exacerbating lactic acidosis in hypoperfused patients have historically limited their widespread adoption in DKA, despite a lack of robust evidence supporting such concerns.

The goal of this systematic review was to assess the clinical outcomes of fluid resuscitation in patients with DKA using BCs versus plain saline, given the ongoing uncertainty regarding the best fluid for DKA resuscitation and the growing but contradictory data from recent trials.

## Review

Methodology

This systematic review was carried out in accordance with the Preferred Reporting Items for Systematic Reviews and Meta-Analyses (PRISMA) 2020 statement [6]. 

Search Strategy

Using internet databases including PubMed/MEDLINE, Scopus, Embase, and the Cochrane Central Register of Controlled Trials (CENTRAL), a comprehensive search of the literature was carried out. In order to gather the most recent and relevant data, the search was limited to the last five years due to evolving clinical guidelines and the recent rise in publications comparing BCs with NS in DKA. The search approach combines free-text keywords and Medical Subject Headings (MeSH), such as: "diabetic ketoacidosis," "DKA," "balanced crystalloids," "balanced electrolyte solutions," "Ringer's lactate," "lactated Ringer," "Plasma-Lyte," "Sterofundin," "normal saline," "0.9% saline," "fluid resuscitation," and "intravenous fluids." Terms were combined using Boolean operators (AND, OR). To find any further relevant research, the reference lists of the included studies and pertinent review articles were manually searched. Only English-language publications were included in the search.

The full Boolean search string for PubMed/MEDLINE was as follows: (`"Diabetic Ketoacidosis"[Mesh] OR "diabetic ketoacidosis"[tiab] OR DKA[tiab]) AND ("Balanced Crystalloids"[tiab] OR "balanced electrolyte solutions"[tiab] OR "Ringer's Lactate"[tiab] OR "lactated Ringer"[tiab] OR "Plasma-Lyte"[tiab] OR "Sterofundin"[tiab] OR "Isolene"[tiab]) AND ("Normal Saline"[tiab] OR "0.9% saline"[tiab] OR "sodium chloride"[tiab]) AND ("fluid resuscitation"[tiab] OR "intravenous fluids"[tiab] OR "fluid therapy"[tiab]).

This search strategy was adapted for Scopus, Embase, and CENTRAL using equivalent controlled vocabulary and syntax. The search yielded a total of 554 records across all databases prior to duplicate removal: PubMed (n=160), Scopus (n=130), Embase (n=184), and CENTRAL (n=80). Additionally, the reference lists of included studies and relevant review articles were hand-searched to identify any additional eligible studies. The search was restricted to articles published in English.

Eligibility Criteria

According to the Population, Intervention, Comparator, Outcomes, and Study Design (PICOS) framework, studies were deemed eligible for inclusion provided they fulfilled the following requirements: Population (P): Patients of any age diagnosed with DKA, encompassing type 1 and type 2 diabetes, as well as any severity (mild, moderate, or severe) as determined by the original research criteria. Intervention (I): Intravenous fluid resuscitation using a BCs solution, such as Sterofundin, which has an electrolyte composition more similar to human plasma than 0.9% saline. Balanced electrolyte solutions (BES) include RL, LR solution, Plasma-Lyte 148, and Isolyte. Comparator (C): Intravenous fluid resuscitation using 0.9% NS (sodium chloride solution). Outcomes (O): Studies had to report at least one of the following outcomes: serum chloride or bicarbonate levels, incidence of hyperchloremia, length of insulin infusion, length of hospital or intensive care unit (ICU) stay, incidence of AKI, need for renal replacement therapy (RRT), time to DKA resolution (defined by anion gap closure, pH ≥7.30, or bicarbonate normalization), or death. Study design (S): Prospective interventional studies, retrospective cohort studies, quasi-randomized trials, and randomized controlled trials (RCTs) that directly compared BCs and NS.

Exclusion criteria were (1) studies (such as single-arm studies, case series, and case reports) that lacked a direct comparator group; (2) studies comparing fluid regimens other than BCs versus NS (e.g., one-bag versus two-bag methods, different volumes of the same fluid); (3) studies using non-standard BCs such as meglumine sodium succinate (Reamberin) due to their distinct metabolic properties; (4) review articles, editorials, commentaries, protocols without published results, and surveys; (5) non-English publications; (6) duplicate publications of the same patient cohort.

Study Selection

All of the retrieved data were imported into the web-based collaborative systematic review management tool Rayyan Intelligent Systematic Review (Rayyan Systems Inc., Cambridge, MA, USA) [7]. Rayyan's duplicate detection feature was used to automatically eliminate duplicate records, which were then manually verified. Two unidentified, unbiased reviewers checked the abstract and title for eligibility. The same two reviewers then obtained potentially relevant full-text publications and independently evaluated them for inclusion. Any disagreements were resolved through discussion or, if necessary, by consulting a third reviewer. The selection process was documented using a PRISMA flow diagram. The search and selection process included studies that were published more than five years ago. Nine studies met the inclusion criteria after this process and were included in the final systematic review.

Data Extraction

A standardized data extraction form was previously created using Microsoft Excel (Microsoft Corporation, Redmond, WA, USA). Two reviewers independently extracted the following data from each included study: sample size (total, NS group, BC group), study design, place of origin, year of publication, first author name, population characteristics (age, sex, DKA severity definition), type of BC used, primary and secondary outcome definitions and results (including exact numerical values such as means, medians, standard deviations, interquartile ranges, hazard ratios, p-values, and confidence intervals whenever reported), information for the risk of bias (ROB) assessment, and the length of follow-up. Any information that was missing was noted as "not mentioned" (NM). Reexamining the original papers and reaching a consensus helped resolve discrepancies in data extraction. To facilitate subgroup analyses, data from pediatric trials were extracted independently.

Risk of Bias Assessment

The ROB in the included studies was assessed using validated tools appropriate for the research design. RCTs were assessed using the Cochrane RoB 2 tool [8]. Five aspects of bias are evaluated by this tool: (1) the randomization process; (2) deviations from intended interventions; (3) inadequate outcome data; (4) outcome measurement; and (5) the selection of the reported result. Each domain was categorized as "low risk," "some concerns," or "high risk," resulting in an overall risk-of-bias judgment.

Non-randomized studies were assessed using the Risk of Bias in Non-randomized Studies of Interventions (ROBINS-I) tool [9]. Bias arising from confounding, participant selection, intervention categorization, deviations from intended interventions, missing data, outcome assessment, and selection of reported findings was assessed by this instrument. An overall evaluation was conducted after each domain was rated as "low," "moderate," "serious," or "critical" risk. Two reviewers performed each evaluation independently, and disagreements were resolved through discussion.

Data Synthesis and Analysis

The included studies exhibited significant clinical and methodological variability, including variations in study design (RCTs versus retrospective cohorts), study populations (adults versus children), definitions of DKA resolution, and the types of BCs used. Instead of doing a meta-analysis, a narrative (descriptive) synthesis was carried out, taking into account the types of BC utilized and the forms for reporting the results. Findings were organized by outcome domain (time to DKA resolution, electrolyte parameters, insulin duration, LOS, renal outcomes, mortality) and by population (adults vs. children) to facilitate interpretation. Results were summarized in two comprehensive tables. 

Results

**Figure 1:**
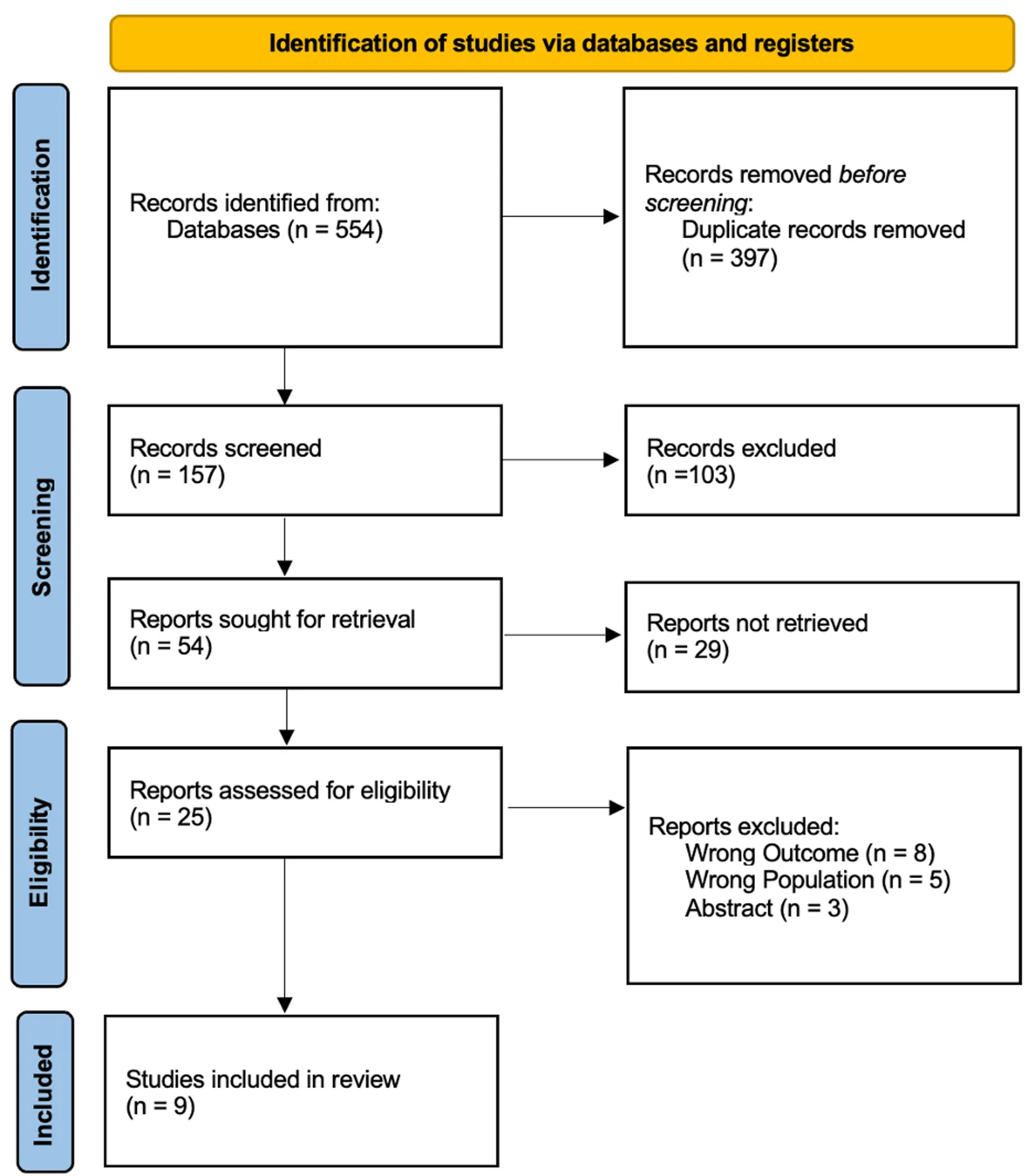
PRISMA flow diagram of the study selection process PRISMA, Preferred Reporting Items for Systematic Reviews and Meta-Analyses

The PRISMA flow diagram outlines the systematic screening and selection process for studies included in the review. A total of 554 records were initially identified from the databases. After removing 397 duplicate records, 157 records proceeded to screening, of which 103 were excluded at the title/abstract stage. Of the remaining 54 reports sought for retrieval, 29 could not be obtained, leaving 25 full-text reports assessed for eligibility. At this final stage, 16 reports were excluded due to the wrong outcome (n=8), the wrong population (n=5), or being available only as abstracts (n=3). Ultimately, nine studies met the inclusion criteria and were included in the final review (Figure 1).

Table 1 outlines the salient features of the nine studies included in this systematic review, which compare BCs to NS for fluid resuscitation in DKA. The studies span from 2022 to 2026 and originate from four countries: Turkey [10], the United States [11-13], Canada [14], and India [15-18]. Four studies are RCTs, namely Yan [14] (pilot triple-blind), Agarwal [16] (double-blind), Singhal [17] (triple-blind), and Sweety [18] (double-blind), while the remaining five are nonrandomized studies, including retrospective cohorts and one prospective interventional trial with historical controls [10-13,15]. Sample sizes vary considerably, from the smallest pilot RCT of 52 patients to the largest retrospective cohort of 771 patient encounters; the pediatric RCTs range from 50 to 67 participants [13,14,16-18]. Regarding the study populations, four studies exclusively enrolled adults, three focused on children, one included patients aged 13 years and older, and one did not specify age [10-18]. BC types included LR (used in six studies [11-14,16,17]), Isolene or LR [10], Sterofundin [15], and a general BES [18]. Notably, most studies did not report sex distribution except Bharti et al. [15] (56.7% male) and Sweety et al. [18] (26.7% male, 73.3% female). The severity of DKA was explicitly stated as moderate-to-severe in Aslan [10] and defined by ISPAD criteria in the pediatric RCTs; other studies did not specify severity [16-18]. Missing demographic data are marked as "NM" (not mentioned).

**Table 1:** Study characteristics and demographic data NS, normal saline (0.9% sodium chloride) group; BC, balanced crystalloid group; NM, not mentioned / not reported; ICU, intensive care unit; DKA, diabetic ketoacidosis; ED, emergency department; PICU, pediatric intensive care unit; PGIMER, Post Graduate Institute of Medical Education and Research (Chandigarh, India); ISPAD, International Society for Pediatric and Adolescent Diabetes; BES, balanced electrolyte solution; T1DM, type 1 diabetes mellitus; PED, pediatric emergency department; RCT, randomized controlled trial; LR, lactated Ringer‘s; RL, Ringer‘s lactate

Study (author, year)	Location	Study design	Sample size (Total)	Sample size (NS)	Sample size (BC)	Population/age	Sex (% male)	DKA severity	BC type	Key demographic notes
Aslan (2025) [10]	Turkey (NM)	Retrospective cohort	80	31	49	Adults in ICU; NM age	NM	Moderate-to-severe	Isolene or LR	NM
Johnson (2024) [11]	USA (single institution)	Retrospective cohort	246	119	127	Adults; NM age	NM	DKA (criteria NM)	LR	Protocol change study
Carrillo (2022) [12]	USA (single-center)	Retrospective cohort	NM	NM	NM	Adults with DKA; NM age	NM	DKA (criteria NM)	LR	Exact sample size not reported
Jamison (2024) [13]	USA (multicenter, single health system)	Retrospective cohort	771	NM	NM	Adults; NM age	NM	DKA (criteria NM)	LR	Largest cohort; encounter-based
Yan (2024) [14]	Canada (London, Ontario; academic ED)	Pilot triple-blind RCT	52	27	25	Adults ≥18 years; median age NM	NM	DKA (by criteria)	RL	Feasibility pilot
Bharti (2025) [15]	India (Chandigarh, PGIMER)	Prospective interventional with historical controls	150	75	75	Age ≥13 years; mean 36.8±NM	56.7%	DKA (criteria NM)	Sterofundin	Historical controls (NS)
Agarwal (2025) [16]	India (teaching hospital, PED/PICU)	Double-blind RCT	67	34	33	Children 9 mo-12 y (T1DM); mean age NM	NM	DKA (ISPAD criteria)	RL	Pediatric only
Singhal (2025) [17]	India (New Delhi, tertiary PICU)	Triple-blind RCT	50	25	25	Children 6 mo-8 y; median 9 (5-12) y	NM	DKA (ISPAD criteria)	RL	Pediatric only
Sweety (2026) [18]	India (tertiary care teaching hospital)	Double-blind RCT	60	~30	~30	Children 5-14 y; mean 10.7±1.42 y	26.7% (73.3% female)	DKA (ISPAD criteria)	BES	Pediatric only

Table 2 compares the clinical results of the BC and NS groups in the nine included studies. Time to DKA resolution, the main endpoint, produced inconsistent findings. A statistically significant faster resolution with BCs was found in four studies: Johnson [11] (mean 17.1 vs. 20.6 hours, p=0.02), Jamison [13] (adjusted HR 1.325, p<0.001), Bharti [15] (13.8 vs. 18.1 hours, p<0.001), and Agarwal [16] (mean difference 3.85 hours, 95% CI 0.3-8). Conversely, four studies found no significant difference: Aslan [10] (median 9 vs. 12 hours, p=0.539), Yan [14] (median 15.7 vs. 12.7 hours, non-significant), Singhal [17] (8 vs. 12 hours, p=0.16), and Sweety [18] (p=0.16). Regarding secondary outcomes, hyperchloremia was consistently lower with BCs wherever reported; Aslan [10] documented significantly lower post-resolution chloride levels (110.8 vs. 115.8 mmol/L, p<0.001), Carrillo [12] reported lower rates of iatrogenic hyperchloremia (64.2% vs. 74.4%, p=0.05), and both pediatric RCTs [16,17] noted reduced chloride increases with RL. Insulin infusion duration was significantly shorter with BCs in Johnson [11] (16.0 vs. 21.4 hours, p<0.001) and Bharti [15] (98 vs. 112 units of insulin, p=0.017), but Jamison [13] reported no difference. Hospital or ICU LOS showed mixed results: Aslan [10] found no difference (44 vs. 46 hours, p=0.961), Singhal [17] reported a shorter pediatric intensive care unit (PICU) stay with LR (p<0.05), and Bharti [15] showed a shorter hospital stay with Sterofundin (4 vs. 4 days, p=0.020). AKI incidence was comparable across studies [12,13,16,18], although Sweety [18] noted a non-significant trend toward higher AKI incidence with NS (p=0.07). Mortality was similar between groups in the two studies reporting it [10,15].

**Table 2:** Clinical outcomes: BCs versus NS DKA, diabetic ketoacidosis; NS, normal saline (0.9% sodium chloride) group; BC, balanced crystalloid group; Cl, chloride; K, potassium; ICU, intensive care unit; LOS, length of stay; AKI, acute kidney injury; RRT, renal replacement therapy; LR, Lactated Ringer's (equivalent to RL); SF, Sterofundin (a proprietary balanced crystalloid solution); HAGMA, high anion gap metabolic acidosis; CI, confidence interval; HR, hazard ratio; RQ, rapport de cotes (French for odds ratio, presented here as adjusted odds ratio); HCO₃, bicarbonate; Cr, creatinine; ΔCr, change in creatinine; RL, Ringer's Lactate (equivalent to LR); PICU, pediatric intensive care unit; BES, balanced electrolyte solution (used in Sweety 2026); NM, not mentioned/not reported.

Study (author, year)	Primary outcome (time to DKA resolution)	Secondary outcome: hyperchloremia/electrolytes	Secondary outcome: insulin infusion duration	Secondary outcome: hospital/ICU LOS	Secondary outcome: AKI/renal function	Secondary outcome: mortality/other
Aslan (2025) [10]	No significant difference: median 12 h (NS) vs. 9 h (BC); p=0.539	Lower Cl in BC: post-resolution Cl 115±5.5 (NS) vs. 110.8±4.4 (BC); p<0.001; K 3.4 vs. 3.6; p=0.088	NM	ICU stay: 46 h (NS) vs. 44 h (BC); p=0.961	NM	1-month mortality: 0% (NS) vs. 4.1% (BC); p=0.524; RRT: 3.2% vs. 4.1%; p=1.000
Johnson (2024) [11]	Significantly shorter with LR: mean 20.6±12.2 (NS) vs. 17.1±11.0 (LR) h; p=0.02	NM	Shorter with LR: 21.4±12.5 (NS) vs. 16.0±8.7 (LR) h; p<0.001	NM	NM	NM
Carrillo (2022) [12]	NM	Reduced hyperchloremia with LR: max Cl 115.7 vs. 113.7; p=0.004; hypernatremia 18.3% vs. 9.3%; p=0.02; 74.4% (NS) vs. 64.2% (LR); p=0.05	NM	No significant difference in ICU or hospital LOS (exact NM)	Better creatinine improvement with LR: ΔCr at 48h -0.04 (NS) vs. -0.15 (LR) mg/dL; p=0.002; AKI incidence no difference	NM
Jamison (2024) [13]	Faster HAGMA resolution with LR: RQ ajustée 1.325 (IC 95% 1.121-1.566); p<0.001	No difference in non-gap metabolic acidosis, hyperchloremia	No difference in insulin duration	No difference in ICU or hospital LOS	No difference in AKI or new RRT	NM
Yan (2024) [14]	No significant difference: median 12.7 (7.9-19.2) NS vs. 15.7 (10.4-18.8) LR h	NM	NM	NM	No major adverse kidney events (composite)	No in-hospital death; no blinding breaks
Bharti (2025) [15]	Faster with Sterofundin: mean 18.1±5.5 (NS) vs. 13.8±6.0 (SF) h; p<0.001	NM	Less insulin with SF: 112 vs. 98 units; p=0.017	Shorter hospital stay with SF: median 4 (4-6) vs. 4 (3-5) days; p=0.020	NM	Mortality similar: 8.1% (NS) vs. 9.3% (SF); p=0.791
Agarwal (2025) [16]	Faster with RL: mean 16.8±9 (NS) vs. 12.9±7.9 (RL) h; mean diff 3.85 h (95% CI 0.3-8); HR=1.39	Lower Cl rise with RL at 4h (8.7 vs. 3.9) and 8 hours (10.8 vs. 4.4); greater HCO_3_ increase with RL at 12 hours (14.7 vs. 12.9).	NM	NM	No difference in AKI incidence	NM
Singhal (2025) [17]	No significant difference: median 12 (4-18) NS vs. 8 (4-10) RL h; p=0.16	Higher hyperchloremia in NS (p<0.05)	NM	Longer PICU stay with NS (p<0.05); total hospital stay similar	Comparable metabolic profile	Comparable complications
Sweety (2026) [18]	No significant difference: p=0.16	Higher hyperchloremia with NS (P=0.09, NS)	NM	Prolonged hospitalization with NS (P=0.23, NS)	Higher AKI with NS (P=0.07, NS)	No difference in cerebral edema

Table 3 provides the ROB assessment for each of the nine included studies using the most appropriate validated tool based on research design. None of the studies was considered to be at critical risk; however, sensitivity analyses were recommended to assess the impact of higher-risk studies. Each study provided valuable information and was included in the systematic review.

**Table 3:** Evaluation of included studies' ROB RCT, randomized controlled trial; ROBINS-I, Risk of Bias in Non-randomized Studies of Interventions; RoB, risk of bias

Study (author, year)	Study design	Assessment tool	Randomization/confounding	Allocation/participant selection	Blinding/intervention classification	Missing data	Outcome measurement	Selective reporting	Overall ROB
Aslan (2025) [10]	Retrospective cohort	ROBINS-I	Serious (confounding by severity)	Moderate	Moderate	Low	Low	Low	Moderate
Johnson (2024) [11]	Retrospective cohort	ROBINS-I	Serious (confounding by time/protocol change)	Moderate	Moderate	Low	Low	Low	Moderate
Carrillo (2022) [12]	Retrospective cohort	ROBINS-I	Serious (unmeasured confounders, unclear sample size)	Serious	Moderate	Serious (missing sample size)	Low	Moderate	Serious
Jamison (2024) [13]	Retrospective cohort (multicenter)	ROBINS-I	Moderate (adjusted for covariates)	Low	Low	Low	Low	Low	Moderate
Yan (2024) [14]	Pilot triple-blind RCT	RoB 2	Low	Low	Low	Low	Low	Some concerns (pilot, no pre-specified analysis plan)	Some concerns
Bharti (2025) [15]	Prospective interventional with historical controls	ROBINS-I	Serious (confounding by time/historical controls)	Moderate	Moderate	Low	Low	Low	Serious
Agarwal (2025) [16]	Double-blind RCT	RoB 2	Low	Low	Low	Low	Low	Low	Low
Singhal (2025) [17]	Triple-blind RCT	RoB 2	Low	Low	Low	Low	Low	Low	Low
Sweety (2026) [18]	Double-blind RCT	RoB 2	Low	Low	Some concerns (blinding of assessors unclear)	Low	Low	Low	Low

Using the Grading of Recommendations, Assessment, Development, and Evaluations (GRADE) approach in Table 4, the certainty of evidence varied substantially across outcomes. For the primary outcome, time to DKA resolution, the certainty was rated as low (⊕⊕◯◯). This was downgraded for serious ROB (predominantly from nonrandomized studies), serious inconsistency (conflicting results between studies), and serious imprecision (wide confidence intervals, small sample sizes in RCTs). For biochemical outcomes (hyperchloremia and bicarbonate), the certainty was moderate (⊕⊕⊕◯), reflecting consistent findings despite some ROB. The certainty for insulin duration, LOS, and mortality was very low (⊕◯◯◯) due to sparse data and inconsistent results. The certainty for AKI was low (⊕⊕◯◯). These GRADE assessments underscore the limited confidence in the current evidence base and highlight the need for further high-quality research before strong clinical recommendations can be made.

**Table 4:** GRADE assessment of certainty of evidence for key outcomes LOS, length of stay; AKI, acute kidney injury; GRADE, Grading of Recommendations, Assessment, Development, and Evaluations; ROB, risk of bias; RCT, randomized controlled trial

Outcome	Number of studies (design)	ROB	Inconsistency	Indirectness	Imprecision	Publication bias	Certainty	Justification
Time to DKA resolution	9 (4 RCTs, 5 non-RCTs)	Serious (-1)	Serious (-1)	Not serious	Serious (-1)	Undetected	⊕⊕◯◯Low	Inconsistent results, heterogeneity, and imprecision in RCTs
Hyperchloremia reduction	8 studies	Serious (-1)	Not serious	Not serious	Not serious	Undetected	⊕⊕⊕◯Moderate	Consistent findings but observational studies contribute
Bicarbonate improvement	7 studies	Serious (-1)	Not serious	Not serious	Not serious	Undetected	⊕⊕⊕◯Moderate	Consistent biochemical benefit across studies
Insulin infusion duration	3 studies	Serious (-1)	Serious (-1)	Not serious	Serious (-1)	Undetected	⊕◯◯◯Very Low	Limited data, inconsistent findings
LOS	5 studies	Serious (-1)	Serious (-1)	Not serious	Serious (-1)	Undetected	⊕◯◯◯Very Low	Mixed results, underpowered
AKI	5 studies	Serious (-1)	Not serious	Not serious	Serious (-1)	Undetected	⊕⊕◯◯Low	Limited events, imprecise estimates
Mortality	2 studies	Serious (-1)	Not serious	Not serious	Serious (-2)	Undetected	⊕◯◯◯Very Low	Rare outcome, insufficient power

Discussion

Across the nine included studies encompassing both adult and pediatric populations, our review demonstrates that BCs offer consistent advantages in reducing hyperchloremia and improving electrolyte profiles. However, the impact on the key clinical outcome, time to DKA resolution, is still unclear and seems to vary depending on the population, research design, and the specific BC used. These nuanced findings are largely consistent with, but also extend, the conclusions of several recently published meta-analyses and systematic reviews on this topic.

One of the largest and most contemporary meta-analyses of RCTs to date was published by Liu et al. [19] in Frontiers in Endocrinology (May 2024). This meta-analysis included 11 RCTs involving 753 patients with DKA. The time to resolution of DKA did not significantly differ between BCs and NS (mean difference -1.49 hours, 95% CI -4.29 to 1.31, P=0.30, I²=65%). Eleven RCTs totaling 753 DKA patients were included in this meta-analysis [19]. There was no significant variation in the incidence of hypokalemia (RR 0.80, 95% CI 0.43 to 1.46) or major adverse renal events (RR 0.88, 95% CI 0.58 to 1.34), according to the same investigators [19]. Our review partially aligns with these findings: among the four RCTs included in our analysis, two pediatric RCTs and one adult pilot RCT demonstrated that the time to DKA resolution did not differ statistically significantly between BCs and NS [14,16,17]. Nevertheless, the BC (RL) versus NS in adults with DKA in the emergency department (BRISK-ED) pilot study by Yan et al. [14] was the only adult RCT included in our review and reported median times of 15.7 hours for RL versus 12.7 hours for NS, a difference that was not statistically significant, likely as a result of the pilot design and the limited sample size.

Crucially, our review included several large retrospective cohort studies that were not captured in the Liu et al. [19] meta-analysis, which was restricted to RCTs. For instance, the LR versus NS in the management of acute DKA (RINSE-DKA) study by Jamison et al. [13], a multicenter retrospective cohort of 771 patients, showed that LR demonstrated a significantly faster time to resolution of high anion gap metabolic acidosis (HAGMA) (adjusted HR 1.325, 95% CI 1.121-1.566, p<0.001). Likewise, Johnson et al. [11] showed a significant reduction in time to DKA resolution from 20.6 hours with NS to 17.1 hours with LR (p=0.02) in a cohort of 246 patients. Similarly, the prospective interventional trial by Bharti et al. [15] showed that Sterofundin, a BC, significantly reduced the mean time to DKA resolution (13.8 vs. 18.1 hours, p<0.001). These results imply that although the combined RCT data may not definitively show that BCs are preferable for DKA resolution, especially in adult populations, the larger observational and quasi-experimental studies consistently favor BCs. This disparity probably results from the shortcomings of current RCTs, many of which lack the power to identify small but clinically significant variations in resolution time.

In contrast to the neutral finding of Liu et al. [19], a previous meta-analysis and systematic review by Alghamdi et al. [20], published in Critical Care Explorations (2022), reported that utilizing NS rather than BCs was associated with a longer time to DKA resolution (mean difference 3.51 hours longer; 95% CI 0.90 to 6.12; moderate certainty), higher post-resuscitation serum chloride, lower post-resuscitation serum bicarbonate, and a longer hospital stay (mean difference 0.89 days longer; moderate certainty) [20]. The use of BCs rather than saline for fluid resuscitation in DKA was supported by this meta-analysis, which comprised eight RCTs with 482 participants and assessed the certainty of the evidence as low to moderate [20]. Our research is more consistent with the findings of Alghamdi et al. [20], particularly when considering the totality of evidence, including both RCTs and high-quality observational studies. Notably, Shaban et al. [21] carried out a more recent systematic review and meta-analysis (April 2025), which comprised six studies with 5033 patients and found no significant difference in the time to DKA resolution between LR and NS (mean difference (MD) 1.12 hours, 95% CI -0.69 to 2.93, p=0.23). However, the LR group had significantly better post-resuscitation bicarbonate and chloride profiles and a significantly shorter ICU stay (MD 4.79 hours shorter, p=0.0008) [21]. This pattern of discordant findings across meta-analyses underscores the heterogeneity in study design, definitions of DKA resolution, and patient populations that characterize the current evidence base.

A more consistent finding across all prior meta-analyses and our own review is the beneficial effect of BCs on serum chloride and bicarbonate levels. Liu et al. [19] found that post-resuscitation chloride levels were significantly lower with BCs (mean difference -3.16 mmol/L, 95% CI -5.82 to -0.49, P = 0.02, I² = 73%) [19]. Alghamdi et al. [20] reported that while the impact on chloride was less definite (95% CI -0.40 to 3.64; moderate certainty; mean difference 1.62 mmol/L greater), patients receiving saline probably had lower serum bicarbonate levels after resuscitation (mean difference -1.50 mmol/L, 95% CI -2.33 to -0.67; moderate certainty) [20]. These results are strongly supported by our review. Aslan et al. [10] showed that the balanced group had significantly lower post-resolution chloride levels (110.8 vs. 115.8 mmol/L, p<0.001). Carrillo et al. [12] reported a lower mean maximum serum chloride level (113.7 vs. 115.7 mmol/L, p=0.004) and a significantly reduced incidence of iatrogenic hyperchloremia with LR (64.2% vs. 74.4%, P=0.05). In the pediatric RCT by Agarwal et al. [16], the LR group showed a significantly lower increase in chloride from baseline at both four hours (3.9 vs. 8.7 mmol/L) and eight hours (4.4 vs. 10.8 mmol/L), as well as a significantly greater increase in bicarbonate after 12 hours (14.7 vs. 12.9 mmol/L). Singhal et al. [17] and others also reported a significantly higher incidence of hyperchloremia in the NS group (p<0.05). These consistent findings across multiple studies and populations establish hyperchloremia reduction as a well-supported benefit of BCs in DKA.

The clinical significance of reducing hyperchloremia in DKA warrants consideration. Hyperchloremic metabolic acidosis can complicate the assessment of DKA resolution, as the anion gap may normalize while a non-anion gap metabolic acidosis persists. This phenomenon was observed by Messina et al. [22] (a study excluded from our primary analysis due to the lack of a BC comparator), who found that following DKA resolution, the incidence of non-anion gap acidosis was significantly higher in patients who received more than two liters of NS [22]. By reducing the chloride load, BCs may facilitate a more complete and physiologically appropriate resolution of acidosis, potentially reducing the risk of diagnostic confusion and unnecessary therapeutic prolongation. A recent systematic review by Ismail et al. [23] (August 2025) found that BCs consistently shortened the time to DKA resolution by 20%-30% and reduced hyperchloremic metabolic acidosis, with chloride differences of 5-8 mmol/L and bicarbonate increases of 2-3 mmol/L [23]. A narrative review of Plasma-Lyte in DKA resuscitation by Sudha (2024) also emphasized the unique capacity of BCs to prevent hyperchloremic metabolic acidosis because their electrolyte composition closely resembles that of human plasma [24].

AKI and other renal outcomes are still being studied in relation to BCs. In the meta-analysis by Liu et al. [19], there was no significant difference between NS and BCs in major adverse renal events (RR 0.88, 95% CI 0.58 to 1.34, P=0.56) [19]. Our study found that there was no significant difference in the incidence of AKI among the studies that reported this outcome. Carrillo et al. [12] found a significantly larger drop in serum creatinine at 48 hours in the LR group (-0.15 vs. -0.04 mg/dL, p=0.002), indicating potentially better renal recovery or less renal impairment with BCs, but found no discernible variation in the incidence of AKI across groups [12]. Jamison et al. [13] found no difference in the incidence of AKI or the need for renal replacement therapy. Agarwal et al. [16] and Sweety et al. [18] also found no discernible variation in the incidence of AKI, although the latter observed a statistically nonsignificant (p=0.07) trend toward higher AKI in the NS group [16,18]. These results are consistent with the Isotonic Solutions and Major Adverse Renal Events Trial (SMART) study and other critical care literature [25]. These studies showed that in critically ill patients, BCs reduced the composite outcome of major adverse kidney events compared with saline, although the magnitude of the effect may be smaller in DKA, a disease that usually affects younger, healthier patients. The absence of a clear signal for renal benefit in DKA may reflect the generally good baseline renal function in this population, the relatively short duration of fluid resuscitation, or insufficient statistical power in existing studies. The ongoing BEST-DKA trial [26] (phase 3 cluster-crossover RCT comparing Plasma-Lyte 148 with saline, expected to enroll ≥400 patients) will provide more definitive evidence on major adverse kidney events and other renal outcomes in the DKA population.

Our review included three pediatric RCTs, providing an important contribution to the literature on BCs in children with DKA [16-18]. These studies, all conducted in India, yielded somewhat variable results. Agarwal et al. [16] showed that using RL considerably reduced the time to DKA resolution (12.9 vs. 16.8 hours, HR 1.39, 95% CI 1.25-1.56). Singhal et al. [17] showed that the NS group had considerably greater hyperchloremia and a longer PICU stay, although there was no significant difference in resolution time (median 8 vs. 12 hours, p=0.16). Sweety et al. [18] also found no discernible variation in resolution time (p=0.16), although this result was probably limited by the study's statistical power. To address these disparities, a recent systematic review and network meta-analysis of fluid regimens in pediatric DKA included studies evaluating RL and Plasma-Lyte [27]. Notably, Ramanan et al. [28] pointed out in their 2024 review that higher fluid administration rates in children have not been associated with cerebral edema, according to cohort studies and randomized trials. However, small sample sizes and single-center designs continue to restrict the pediatric evidence base, and more multicenter RCTs are necessary before definitive recommendations for this population can be established.

An important consideration in interpreting the evidence is the heterogeneity of BC formulations used across studies. Our review captured studies using LR, Isolene or LR, Sterofundin, and a general BES [10-18]. While all these solutions share the characteristic of having chloride concentrations closer to that of human plasma compared with NS (154 mmol/L), they differ in their specific electrolyte composition, buffers, osmolality, and buffer constituents (acetate and gluconate in Plasma-Lyte, succinate in Sterofundin, and lactate in LR). Liu et al.'s meta-analysis [19] found no discernible subgroup variations according to the type of BC, indicating that the class effect could be comparable across different formulations. However, the study by Bharti et al. [15] using Sterofundin demonstrated particularly robust benefits, including shorter DKA resolution (13.8 vs. 18.1 hours), lower total fluid requirements (4500 vs. 6000 mL), and lower insulin requirements (98 vs. 112 units) [15]. A dedicated commentary on Sterofundin in DKA noted that this succinate-containing solution may offer additional metabolic advantages through the metabolism of succinic acid, potentially accelerating acid-base normalization [17]. Similarly, Plasma-Lyte has been the subject of ongoing investigation. The BEST-DKA trial is expected to provide high-quality evidence specifically for Plasma-Lyte 148, while a narrative review by Alghamdi (2024) summarized the existing evidence on Plasma-Lyte in DKA resuscitation and found no discernible subgroup differences according to the type of BC, suggesting that the class effect may be comparable across different formulations [24,26].

The current evidence base, as assessed by GRADE, provides moderate certainty for biochemical benefits (chloride and bicarbonate improvement) but low to very low certainty for clinically meaningful outcomes. While biochemical advantages are consistent, the clinical relevance of reducing hyperchloremia in DKA, particularly when it does not consistently translate to faster resolution or shorter hospital stay, remains uncertain. Clinicians may reasonably choose BCs to avoid iatrogenic hyperchloremia but should interpret this as a preference based on secondary endpoints rather than a practice-defining recommendation supported by high-certainty evidence. The ongoing BEST-DKA trial is expected to provide higher-certainty evidence that will inform future guideline development.

Study Limitations

Several limitations of this systematic review include substantial heterogeneity in study designs, varying definitions of DKA resolution, and a predominance of single-center studies that may limit generalizability. The lack of blinding in retrospective cohort studies poses a potential ROB, while small sample sizes, especially in larger studies, raise concerns about confounding. Additionally, long-term outcomes after discharge remain unreported, indicating a significant knowledge gap. Lastly, there is a potential for publication bias, although the inclusion of neutral studies suggests that this may be minimal.

## Conclusions

BCs reduce hyperchloremia and improve bicarbonate profiles compared with NS in patients with DKA, without increasing adverse events. However, the effect on clinically meaningful outcomes, particularly time to DKA resolution, duration of insulin infusion, and LOS, remains uncertain due to inconsistent findings and low-certainty evidence. Based on the GRADE assessment, the certainty of evidence is moderate for biochemical endpoints but low to very low for patient-important outcomes. While the biochemical advantages and consistent safety profile support considering BCs as an alternative to NS, the current evidence does not justify a definitive recommendation for BCs to become the standard of care. High-quality, adequately powered trials such as BEST-DKA are urgently needed to determine whether the biochemical benefits translate into improved clinical outcomes.
